# Prevalence and factors associated with major depression among female sex workers in post-conflict Gulu district: a cross-sectional study

**DOI:** 10.1186/s12889-021-11207-8

**Published:** 2021-06-13

**Authors:** Simple Ouma, Nazarius Mbona Tumwesigye, Rawlance Ndejjo, Catherine Abbo

**Affiliations:** 1 Department of Research, The AIDS Support Organization (TASO), Kampala, Uganda; 2 Department of Epidemiology and Biostatistics, School of Public Health, College of Health Sciences, Makerere University, Kampala, Uganda; 3 Department of Disease Control and Environmental Health, School of Public Health, College of Health Sciences, Makerere University, Kampala, Uganda; 4 Department of Psychiatry, School of Medicine, College of Health Sciences, Makerere University, Kampala, Uganda

**Keywords:** Major depression, Female sex workers, Gulu district, Post-conflict setting

## Abstract

**Background:**

Female sex workers operating in conflict-affected settings could be at a much greater risk of major depression. However, the epidemiology of major depression in this population remains understudied. We aimed to determine the prevalence and the factors associated with major depression among FSWs in the post-conflict Gulu district in Northern Uganda.

**Methods:**

We conducted a cross-sectional study among 300 randomly selected adult female sex workers in Gulu. We utilized a pre-tested semi-structured questionnaire, embedded with MINI 7.0.0, to gather information from each participant through face-to-face interviews. We collected data on socio-demographic characteristics, sex-work-related characteristics, alcohol and drug use, HIV status, and major depression. Then, data were entered into EPI INFO 7 and analyzed using logistic regression with the aid of STATA 14.0.

**Results:**

The mean age (SD) of the study participants was 26.4 (± 6) years, 57.7% attained primary education, 51.7% never married, and 42.1% were living with HIV. The prevalence of major depression among FSWs in the district was 47.7%. In addition, the majority of the FSWs with major depression (91.0%) had either severe (50.4%) or moderate (40.6%) depressive symptoms. Independently, life stress (adjusted OR = 10.8, 95%CI: 5.67–20.57), living with HIV (adjusted OR = 2.25, 95%CI: 1.25–4.05), verbal abuse (adjusted OR = 2.27, 95%CI: 1.27–4.08), and older age (adjusted OR = 1.06, 95%CI: 1.01–1.12) all showed positive associations with major depression. Conversely, provision of sexual services from clients’ homes (adjusted OR = 0.50, 95%CI: 0.25–0.97), use of a non-barrier modern family planning method (adjusted OR = 0.44, 95%CI: 0.24–0.82), and daily intake of alcohol (adjusted OR = 0.50, 95%CI: 0.28–0.88) all showed negative associations with major depression.

**Conclusions:**

There is a high prevalence of major depression among female sex workers in post-conflict Gulu. The high prevalence of major depression underscores the need for government and development partners to urgently and adequately address the mental health needs of female sex workers.

**Supplementary Information:**

The online version contains supplementary material available at 10.1186/s12889-021-11207-8.

## Background

Depression is the leading cause of disability and a major contributor to the overall global burden of disease [1]. By 2017, depression was affecting more than 300 million people worldwide [1]. In addition, depression accounts for 32·4% of years lived with disability and 13·0% of disability-adjusted life-years [2]. Individuals with depression incur very high financial costs in terms of treatment, morbidity, and mortality. The annual cost per case of depression is in the range of £3500–£6600 [3]. Depression is a spectrum of chronic disorders with several sub-categories ranging from major depression (MD) to dysthymia [4]. MD, which is the subject of this study, is characterized by depressed mood, loss of interest and enjoyment, and decreased energy and can be graded as mild, moderate, or severe depending on the numbers and severity of symptoms [4].

A recent meta-analysis revealed the need for more studies on MD among female sex workers (FSWs) in the low- and middle-income countries (LMICs) since MD among FSWs in the LMICs remains understudied [5]. The same report indicates that the magnitudes of MD among FSWs vary across countries with the highest prevalence being among FSWs in Jamaica [93.3%] and the lowest prevalence being among FSWs in Bangladesh (4. 2%] [5]. In Kampala, the capital city of Uganda, 43.2% of FSWs have MD [6]. In conflict-affected settings, there are only a handful of studies on MD among FSWs. Yet the negative socio-economic impacts of conflicts and the traumatic life events in conflict settings [7, 8] are well known to increase the risk of MD. A study among Nepalese FSWs points to a very high prevalence of MD (82.4%) in post-conflict settings [9]. Besides conflict, several other factors like chronic physical illnesses, traumatic life events, loss of loved ones, social adversity, extreme poverty, and female gender all can lead to MD [10, 11]. Specifically, the risk factors of MD among FSWs include exposures to various forms of gender-based violence (GBV), psychological and physical burden of sex work [12, 13], as well as alcohol and drug use [14]. The risk of MD among FSWs in Uganda is even greater because of the illegality of sex work [15] that can lead to stigma, workplace violence, GBV, and depression among FSWs.

If left untreated, MD can lead to profound disability, suicide, and other indirect deaths through causing or worsening of physical illnesses [16]. In addition, poorly treated MD can lead to a reduction in sexual satisfaction [17]. Meanwhile, the use of anti-depressants to treat MD can also lead to sexual dysfunction [18]. Moreover, untreated MD among FSWs can impede the progress made towards HIV prevention because it can lead to a reduction in condom use [19]. Likewise, the HIV-positive FSWs with MD are more likely to have poor adherence to their antiretroviral therapy (ART) with resultant unsuppressed viral load and subsequent transmission of HIV infections to their clients or children [20]. To make matters worse, HIV-negative FSWs with MD may have diminished ability to negotiate for safer sex with their clients and become victims of sexual violence like rape and other risky sexual behaviors [21]. Nevertheless, prevention of MD through raising awareness about mental health, early diagnosis, and management can be cost-saving [22]. To generate information needed by the Ugandan health system for the development of robust mental health interventions for FSWs, we aimed to determine the prevalence and the factors associated with MD among FSWs in the post-conflict Gulu district in Northern Uganda.

## Methods

### Study setting, design, and population

We conducted a cross-sectional study among FSWs operating in the post-conflict Gulu district in Uganda. People living in Gulu are still undergoing economic recovery from the more than 20 years of the Lord’s Resistance Army rebellion that devastated their social and economic livelihoods. More than 80% of people in the district practice subsistence farming [23]. An estimated 1425 FSWs operate in the district [24]. The majority of FSWs Gulu live and work in the municipality. In addition, the majority of FSWs receive HIV prevention or treatment services from The AIDS Support Organization (TASO), a national Non-Governmental Organization (NGO) that provides HIV treatment, care, and preventive services across Uganda through its 11 Clinics [25]. TASO provides HIV care and prevention as well as reproductive health services to all the key population groups. TASO conducted mapping for all the key population groups that include the FSWs. TASO Gulu mapped more than 1300 FSWs who operate in the district. The up-to-date database of FSWs contains sex work venues, hang-out places, and their residence. We conducted a cross-sectional study among adult FSWs in the district.

### Sample size and sampling

We calculated the sample size using the Cochran [26] formula: n_0_ = Z^2^pq/e^2^. At 95% confidence level (CI), 5% precision level, and when the proportion of FSWs in Uganda with depressive symptoms is 27% [27], the calculated sample size was 303. We adjusted the sample size by 20% to 380 participants to cater for mobility and non-response. We included all the FSWs aged 18 years and above who had been active in sex work within 6 months before data collection. We identified 789 eligible FSWs from the key populations’ database at TASO Gulu. Then, we utilized an online random number generator to select the 380 participants by simple random sampling technique. We had planned to exclude the FSWs who would be unable to participate because of being bedridden with sickness or mentally incapacitated due to mental illness, alcohol-related disorder, or drug-related disorder. However, none of the selected participants met any of the above exclusion criteria. Meanwhile, to minimize non-response bias, we reached out to the selected FSWs by telephone, through peers, physically traced them using the mapping information at TASO, or met them during clinic days. Out of the 380 selected FSWs, we successfully tracked 302 participants, among whom 300 consented and responded to the questionnaire from conducive places they chose.

### Data collection and management

We collected data between March and June 2020. Using a pre-tested semi-structured questionnaire developed in English and translated into Acholi language, we conducted face-to-face interviews to gather information from participants. We pre-tested the questionnaire among 20 FSWs from the neighboring Amuru district to check for consistency in question interpretation and language appropriateness. Together with a female research assistant, the first author collected participant’s data in either Acholi or English as dictated by participant’s literacy level and preference. Independent variables in this study included: socio-demographic characteristics, sex work-related characteristics, monthly income, place of sex work, sex work-related mobility, alcohol use, illicit drug use, and HIV status. A detailed questionnaire used to assess these independent factors is in Supplementary file 1. The study outcome was MD diagnosed using MINI 7.0.0, a tool based on the Diagnostic and Statistical Manual of Mental Disorders-Fifth Edition (DSM-5) diagnostic criteria [28]. A participant had MD, if she had at least 2 weeks of persistent depressed mood or hopelessness, plus additional symptoms from MD diagnostic criterion A, for a total of 5 of the 9 DSM-5 criteria for MD [29]. Besides, the MD symptoms must have caused significant distress and significantly impaired occupational functionality. MD cases were classified based on severity as; mild if there were 5 symptoms (the minimum needed for MD diagnosis), moderate if there were 6–7 symptoms, and severe if there were 8–9 symptoms [30]. Finally, data were entered and cleaned in EPI INFO 7 and then exported to STATA 14.0 for analysis. We recorded only a few missing socio-demographic data that did not warrant exclusion from data analysis since all participants had provided adequate information to answer the study objectives.

### Statistical analysis

We described univariate results using frequencies with corresponding proportions for categorical variables or means with corresponding standard deviations (SD) for continuous variables. To examined factors associated with MD, we conducted both bivariable and multivariable analyses. Using simple logistic regression, we conducted bivariable data analyses and described its results using the unadjusted odds ratios (OR) with their corresponding CIs and *p*-values. We considered all the significant variables with *p*-values less than 0.20 [31] for inclusion in the multivariable logistic regression [31]. Before running the multivariable logistic regression, we first checked the variables for multicollinearity. We only included one into the multivariable model, whenever two or more variables showed multicollinearity (*r* ≥ 0.4). Thus, we excluded both physical abuse (*r* = 0.90) and rape by clients (*r* = 0.43) since they were multicollinear with verbal abuse. Likewise, we excluded parity (*r* = 0.50) after showing collinearity with age. Then, we entered the remaining independent variables at the beginning step of model building. Utilizing the backward elimination method, we sequentially removed each factor with the least significant *p*-value while testing the model fit using the goodness-of-fit test until we obtained the best fit model. We reported the multivariable results using the adjusted OR with corresponding 95% CIs and *p*-values. Lastly, we executed the regression diagnostics tests on the final multivariable logistic regression model: checked for outliers and influential points using the predicted residuals, conducted Hosmer-Lemeshow’s goodness-of-fit test for the model’s fit, assessed the model’s predictive power using sensitivity and specificity analyses, and finally checked for specification error using the linktest.

## Results

### Participants and participants' socio-demographic characteristics

We successfully tracked 302 out of the 380 randomly sampled participants. Among the 302 participants successfully tracked, 300 consented and were enrolled in the study while two declined to consent for the study - giving a non-response rate of 0.7%. Among the participants: the mean age (SD) was 26.4 (± 6.0) years; 57.7% attained primary education; 51.7% never married; and one-fifth (26.7%) were divorced. The majority of the participants: resided within Gulu (99.4%); resided within urban areas (90.7%); were Catholic (60.7%); and originated from Gulu (48.7%) (Table 1).

**Table 1:** Socio-demographic characteristics of FSWs operating in post-conflict Gulu

Characteristic	Number (***N*** = 300)	Percentage (%)
Age (years)		
Mean (SD)	26.4 (± 6.0)	(18–50)
Education level		
None	13	4.3
Primary education	173	57.7
O-level education	94	31.3
A-level and above	20	6.7
Origin		
Gulu District	146	48.7
Other District within Northern Uganda	125	41.6
Outside Northern Uganda	29	9.7
Location of residence		
Urban	272	90.7
Rural	28	9.3
Marital status		
Never married	155	51.7
Cohabiting	37	12.3
Married	6	2.0
Divorced	80	26.7
Widowed	22	7.3
Religion		
None	18	6.0
Catholic	182	60.7
Protestant	48	16.0
Born Again	34	11.3
Muslim	16	5.3
Others	2	0.7

### Sex work-related characteristics of the study participants

Among the participants; only two-thirds (66.0%) reported consistent condom use, 62.0% were using a non-barrier modern family planning method, 42.1% were living with HIV, and 55.9% were drinking alcohol daily. In addition, 52% of the participants reported verbal abuse, 48.3% reported physical abuse, and up to 62.7% reported feeling stressed during sex work (Table 2).

**Table 2:** Sex work-related characteristics of FSWs operating in post-conflict Gulu

Characteristic	Number (***N*** = 300)	Percentage (%)
Always use condoms		
Yes	198	66.0
No	102	34.0
Use any non-barrier family planning method		
Yes	186	62.0
No	114	38.0
HIV status		
Positive	126	42.1
Negative	173	57.9
Alcohol intake		
None or occasional	131	44.1
Always	166	55.9
Exposed to verbal abuse by clients		
Yes	156	52.0
No	144	48.0
Exposed to physical abuse by clients		
Yes	145	48.3
No	155	51.7
Raped by clients		
Yes	63	21.0
No	237	79.0
Feeling stressed		
Yes	188	62.7
No	112	37.3

### Reasons for joining sex work

**Fig. 1:**
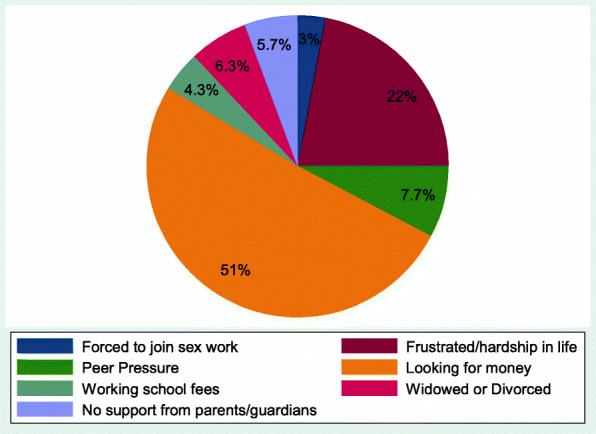
Showing reasons for joining sex work among female sex workers in Gulu

The majority of the participants (89.7%) mentioned poverty-related reasons as their driving factors for joining sex work. Specifically, 51% of FSWs were looking for money for survival, 22% were frustrated by financial hardship, 6.3% were widowed/divorced and lacked financial supports, 5.7% did not have supportive parents or guardians, and 4.3% were looking for school fees (Fig. 1).

### Prevalence of MD among female sex workers

Among the participants, 47.7% met the DSM-5 diagnostic criteria for MD by having: 1) at least 2 weeks of either persistent depressed mood or hopelessness, 2) plus additional symptoms from the MD diagnostic criterion A, for a total of at least five MD symptoms, and 3) the symptoms caused significant distress or problem and significantly altered their behavior or functionality. The majority of MD cases were severe (50.4%) or moderate (40.6%), and only 9.0% of the MD cases were mild. The most common MD symptoms were: feeling depressed (62.3%) and feeling less interested in most things or much less able to enjoy the things one used to enjoy most of the time (59.7%). Also, a sizeable proportion of the participants reported a lack of energy (53%), unintentional changes in appetite or weight (52.2%), and trouble sleeping (50.7%). Meanwhile, the least common symptom of MD was suicidal ideation (16.4%) (Table 3).

**Table 3:** Prevalence of MD among FSWs in post-conflict Gulu District

DSM 5 Diagnostic Criteria for MD	Yes N (%)
1. Have you been consistently depressed or down, most of the day, nearly every day, for the past 2 weeks?	187 (62.3)
2. In the past 2 weeks, have you been much less interested in most things or much less able to enjoy the things you used to enjoy most of the time?	179 (59.7)
Over the past 2 weeks, when you felt depressed or uninterested:	
3. Was your appetite decreased or increased nearly every day? Did your weight decrease or increase without trying intentionally?	156 (52.2)
4. Did you have trouble sleeping (difficulty falling asleep, waking up in the middle of the night, early morning awakening, or sleeping excessively) nearly every night?	151 (50.5)
5. Did you talk or move more slowly than normal, or were you fidgety, restless, or having trouble sitting still almost every day?	154 (51.5)
6. Did you feel tired or without energy almost every day?	159 (53.0)
7. Did you feel worthless or guilty almost every day?	100 (33.1)
8. Did you have difficulty concentrating or making decisions almost every day?	79 (26.3)
9. Did you repeatedly consider hurting yourself, feel suicidal, or wish that you were dead?	49 (16.4)
10. Do symptoms these symptoms cause significant distress or problems at home, at work, socially, in your relationships, or in some other way, and are they a change from previous functioning?	147 (49.0)
Participant meet*s* the *DSM-5 diagnostic criteria for MD*	143 (47.7)
**Severity of MD**	
Mild (had five MD symptoms)	13 (9.0)
Moderate (had 6–7 MD symptoms)	58 (40.6)
Severe (had 8–9 MD symptoms)	72 (50.4)

### Factors associated with MD among female sex workers

Results from the bivariate analysis indicated that several factors had significant associations with MD. Factors that showed positive associations with MD included life stress (*p* < 0.001), living with HIV (*p* < 0.001), parity (*p* < 0.001), older age (*p* < 0.001), verbal abuse by clients (*p* < 0.01), physical abuse by clients (*p* = 0.02), and being raped by clients (*p* < 0.05). Meanwhile, daily alcohol intake (*p* = 0.01) and consistent use of condoms (*p* = 0.04) showed negative associations with MD. At multivariable level, after controlling for several sex work-related variables, factors that remained positively associated with MD were life stress (adjusted OR = 10.8, 95%CI: 5.67–20.57), living with HIV (adjusted OR = 2.25, 95%CI: 1.25–4.05), verbal abuse by clients (adjusted OR = 2.27, 95%CI: 1.27–4.08), and older age (adjusted OR = 1.06, 95%CI: 1.01–1.12). Meanwhile, factors that remained negatively associated with MD were the provision of sexual services from the clients’ homes (adjusted OR = 0.50, 95%CI: 0.25–0.97), use of a non-barrier modern family planning method (adjusted OR = 0.44, 95%CI: 0.24–0.82), and daily intake of alcohol (adjusted OR = 0.50, 95%CI: 0.28–0.88) (Table 4). Lastly, the regression diagnostic tests revealed that the final logistic regression model had no outliers, all the points were evenly distributed across the central lines of residual equal to zero, a good fit test outcome (*p* = 0.55), no specification error (linktest hatsq, *p* = 0.868), and an excellent predictive power (area under the ROC curve = 0.83).

**Table 4:** Bivariate and multivariable analyses of factors associated with MD among FSWs

Factor	Participant has MD		Unadjusted OR	Adjusted OR
	Yes N (%)	No N (%)	(95%CI)	(95%CI)
Age (years)				
Mean (SD)	27.7 (6.4)	25.3 (5.4)	1.07 (1.03–1.12) **	1.06 (1.01–1.12) *
Parity^a^				
0–1	46 (36.8)	79 (63.2)	1.00	–
2	37 (47.4)	41 (52.6)	1.55 (0.87–2.75)	–
3+	60 (61.9)	37 (38.1)	2.78 (1.61–4.82) ***	–
Use family planning^b^				
No	59 (51.8)	55 (48.2)	1.00	1.00
Yes	84 (45.2)	102 (54.8)	0.77 (0.48–1.22) |*|	0.44 (0.24–0.82) **
Always use condom				
No	57 (55.8)	45 (44.2)	1.00	–
Yes	86 (43.4)	112 (56.6)	0.61 (0.42–0.98) *	–
Verbally abused				
No	57 (39.6)	87 (60.4)	1.00	1.00
Yes	86 (55.1)	70 (44.9)	1.88 (1.18–2.97) **	2.27 (1.27–4.08) **
Physically abused				
No	64 (41.3)	91 (58.7)	1.00	–
Yes	79 (54.5)	66 (45.6)	1.70 (1.08–2.69) *	–
Raped by client				
No	106 (44.7)	131 (55.2)	1.00	–
Yes	37 (58.7)	26 (41.3)	1.76 (1.001–3.08) *	–
Does sex work from the clients’ homes				
No	47 (56.0)	37 (44.0)	1.00	1.00
Yes	96 (44.4)	120 (55.6)	0.63 (0.38–1.05) |*|	0.50 (0.25–0.97) *
HIV Status				
HIV-Négative	65 (37.6)	108 (62.4)	1.00	1.00
HIV-Positive	78 (61.9)	48 (38.1)	2.70 (1.68–4.33) ***	2.25 (1.25–4.05) **
Feeling stressed				
No	18 (16.1)	94 (83.9)	1.00	1.00
Yes	125 (66.5)	63 (33.5)	10.4 (5.75–18.66) ***	10.8 (5.67–20.57) ***
Alcohol intake				
None or occasional	73 (55.7)	58 (44.3)	1.00	1.00
Always	68 (40.7)	98 (59.3)	0.55 (0.34–0.87) **	0.50 (0.28–0.88) *

^a^The total number of pregnancies carried to at least 28 weeks of amenorrhoea.

^b^Currently using a non-barrier modern family planning method

|*| = 0.05 < *p* < 0.2, * = *p* < 0.05, ** = *p* < 0.01, *** = *p* < 0.001

## Discussion

Almost half (47.7%) of FSWs in the district had MD. The current magnitude of MD is well above that: in the general population (24.7%) in Gulu [32], among women in the district (29.2%) [32], among FSWs in Southern India (29%) [33], among FSWs in China (31%) [34], and in the general population (10.8%) in conflict-affected settings [35]. However, the current prevalence of MD is comparable to the prevalence of MD among men who have sex with men in Tanzania (46.3%) [36] and the prevalence of MD in the general population in post-conflict South Sudan (50%) [7]. Most FSWs with MD (91.0%) had either severe (50.4%) or moderate (40.5%) depressive symptoms. A similar severity of MD was recorded in the US whereby 89.2% of cases of MD had either severe (49.5%) or moderate (39.7%) depressive symptoms [30]. Moreover, up to 91.0% of FSWs with MD in Gulu require anti-depressants as recommended [35]. This high magnitude of moderate-to-severe cases of MD is a wake-up call to the Ministry of Health and health programmers to urgently develop mental health interventions that screen, prevent, and treat MD among FSWs in the country. In addition, since MD is associated with risky sexual behaviors like condom-less sex, stakeholders need to integrate interventions targeting MD within the existing sexual reproductive health services for FSWs.

Several factors were found to increase the odds of MD among FSWs. Importantly, the odds of MD among FSWs with life stress were 11 times higher than that among FSWs without life stress. This is because, in conflict-affected settings, women are more exposed to life stress since they are the primary caretakers of families and the greatest victims of the conflicting parties [35]. Specifically, the FSWs may get stress from sex work-related violence and the constant fear of being arbitrarily arrested by the police [37]. The current finding is in agreement with a previous report showing that life stress can lead to depression mediated through several biological and social factors like coping strategies [38]. Thus, health programmers should screen FSWs for life stress to ensure early detection of stress. In addition, mental health programmers need to build the capacity of FSWs to cope with life stress and mitigate their vulnerability to MD. It is worth noting that almost nine out of every ten (89.7%) FSWs joined sex work because of poverty. It is well known that many FSWs suffer from depression due to poverty [5]. FSWs who live in poverty are at higher risk of exposure to life stress and need economic empowerment programs. In addition, FSWs who were verbally abused by their clients were almost three times more likely to suffer from MD. This finding is in agreement with studies among FSWs in India [33] and transgender Latinos in Los Angeles [39]. In a country like Uganda where verbal abuse towards FSWs is common [40], FSWs are at higher risk of MD due to acute stress reactions, psychological distress, and anxiety that follows verbal abuse [41]. Further analysis also revealed that FSWs living with HIV were almost three times more likely to suffer from MD than their HIV-negative counterparts. This is in line with a systematic review showing that MD is prevalent among people living with HIV in East Africa [42]. The high prevalent of MD among people living with HIV is not unique to FSWs and is mediated through multiple HIV-related factors like opportunistic infection, perceived HIV-related stigma, hospitalization, and food insecurity among people living with HIV [42]. This increased risk of MD among FSWs living with HIV is alarming because an HIV-positive FSW with MD is unlikely to adhere well to ART resulting in unsuppressed viral load and transmission of HIV infections to clients or infants [20, 43]. Therefore, the Ministry of Health and the health care programmers should consider setting up interventions that provide counseling to FSWs living with HIV to address the several HIV-related factors that put them at greater risk of MD. Lastly, study findings revealed that the odds of MD increase with the participant’s age. This agrees with previous findings from conflict-affected Sri Lanka and other conflict-affected settings [10, 35]. However, in the general population in non-conflict settings, the occurrence of MD did not differ with age [44]. In addition, further analysis indicated that numbers of past pregnancies (*r* = 0.50) showed collinearity with age. The effect of age on MD could be due to the increasing burdens associated with pregnancies and their outcomes on the lives and work of FSWs. Thus, the ministry of health and the health care programmers need to provide targeted mental health prevention programs that best address the mental health needs of the older FSWs. Besides, the presence of multiple factors associated with MD among FSWs underlines the urgent need for multiprong mental health interventions for the FSWs operating in post-conflict settings.

Conversely, FSWs who provided sexual services from the clients’ homes had lower odds of MD. To the best of our knowledge, no previous study ever reported on the relationship between provision of sexual services from the clients’ homes and MD. However, a Canadian study on the relationship between place of sex work and mental illnesses reported that FSWs who provided sexual services in the outdoor/public spaces and the informal indoor spaces were at an increased risk of mental health problems [45]. We postulate that the decrease in GBV among FSWs who provide sexual services from the clients’ homes [40] may be responsible for the reduction in the odds of MD in this sub-group of FSWs. However, there is a need for further studies to understand the exact relationship between MD and specific places of sex work. Secondly, we noted that the use of a non-barrier family planning method significantly reduces the odds of MD among FSWs. Perhaps this is possible because an effective family planning method is known to protect FSWs against unintended pregnancies that would otherwise come with a lot of anxieties and several negative consequences on the life and work of FSWs. This finding agrees with one previous study reporting a reduction of MD among women using family planning [46]. However, others studies did not find any relationship between the use of a family planning method and MD [47, 48]. Yet another study reported mixed outcomes depending on the type of contraception used [49]. Thus, there is a need for more robust longitudinal studies to understand this phenomenon better. Lastly, daily intake of alcohol also reduced the odds of MD among FSWs. At a low level, alcohol may relieve MD symptoms among FSWs because the social nature of alcohol intake can act as a coping strategy against sex work-related stress [50]. However, two longitudinal studies did not find any effect of alcohol on the occurrence of MD [51, 52]. Therefore, much as alcohol appears to reduce MD risk in this study, current finding should be interpreted with caution since alcohol use is known to predispose FSWs to condomless sex [53] that exposes them to sexually transmitted infections, unwanted pregnancy, and induced abortion. Further, chronic and excessive alcohol intake can lead to mental disorders like suicide [54], depression, dementia [55], and can lead to non-recovery from mental disorders [56]. Thus, there is a need for robust studies to understand any causal relationship between MD and alcohol use.

### Strengths and limitations of the study

Unlike most previous studies that used non-probability sampling methods among FSWs, we selected a representative sample of FSWs using a random sampling technique. Thus, current study findings are more generalizable to similar contexts. Also, unlike most previous studies that only screened for depressive symptoms, we diagnosed MD based on DSM-5 criteria. Also, all the regression diagnostic tests showed that the multivariable logistic regression model performed well. However, the study had some limitations. We conducted a cross-sectional study that elicited associations but not causation. Secondly, the information collected may have been influenced by recall bias since we asked FSWs about their past. However, most of the information asked was for the events within 2 weeks, thus reducing the possibility of recall bias. Lastly, some of the implored information relating to sex work was sensitive and difficult to provide. However, the interviewers had close working relationships with the FSWs.

## Conclusions

This current study underscores the high magnitude of MD driven by multiple sex work-related factors like the presence of a psychosocial stressor, living with HIV, experiencing verbal abuse from clients, and older age. The high magnitude of MD cases poses significant public health ramifications at the individual and societal levels. Therefore, there is a need for urgent interventions from the government, the development partners, other health care programmers to address the mental health needs of FSWs in the post-conflict Gulu and beyond. These mental health services may take the form of screening, diagnosis, counseling, and treatment.

## Supplementary Information

**Additional file 1.**

## Data Availability

The datasets used in this study are available from the corresponding author on reasonable request.

## Abbreviations

ART: Antiretroviral Therapy
CI: Confidence Interval
DSM-5: Diagnostic and Statistical Manual of Mental Disorders- Fifth Edition
FSWs: Female Sex Workers
HIV: Human Immunodeficiency Virus
LMICs: Low- and Middle- Income Countries
MD: Major Depression
MINI: Mini-International Neuropsychiatric Interview
NGO: Non-Governmental Organization
OR: Odds Ratios
SD: Standard Deviation
TASO: The AIDS Support Organization
UGX: Ugandan Shillings
UNAIDS: Joint United Nations Program on HIV and AIDS
