# Supplementary material for: Prevalence and factors associated with major depression among female sex workers in post-conflict Gulu district: a cross-sectional study
Source: BMC Public Health. 2021 Jun 13;21:1134. doi: 10.1186/s12889-021-11207-8 (PMC8201688; doi:10.1186/s12889-021-11207-8)

**Questionnaire: English Version**.

**(***Tick inside the box indicating respondent’s answer/ Fill spaces with responses from respondent***)**

- - 1. **Demographic Characteristics**

1. Age in completed years
2. Education level (*formal*)
3. District of Origin: Gulu
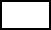
, Others
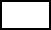
 (specify)
4. District of residence: Gulu
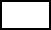
, Others
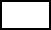
 (specify)
5. Location of current place of residence: Urban
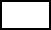
, Rural
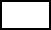

6. Marital Status:

Never married
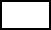
, Cohabitating
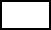
, Married (mono)
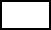
, Married (poly)
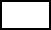
, Separated
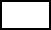
, Widowed
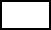


1. Religion: None
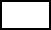
, Catholic
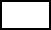
, Protestant
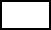
, Born Again
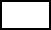
, Muslim
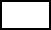
, Others
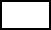
 (specify)
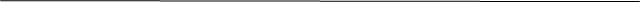

   - 1. **Obstetric History**
2. Age at a first sexual encounter
3. Gravidity
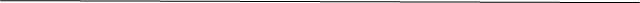

4. Para +
5. If you ever had an abortion, how many were induced abortions?
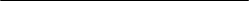

6. If you ever had an induced abortion, did it require post-abortion care?

Yes
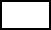
, No
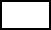


1. How many times did you experience unplanned pregnancy?
2. How many times have you conceived since you started sex work?
3. Besides condoms, are you currently using any modern contraceptives?

Yes
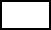
, No
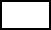


1. If using the modern family planning method, which one?

IUD
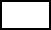
, Implanon
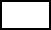
, Injectaplans
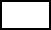
, Bilateral tubal ligation
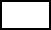
, Oral pills
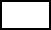
, emergency pills
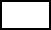


- - 1. **Socio-economic and Sex Work-related characteristics**

1. When did you commence sex work (mm/yyyy)?
2. Why did you join sex work?
3. Were you forced by someone to join sex work? Yes
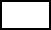
, No
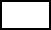

4. What is your estimated monthly income in Uganda Shillings?
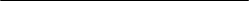

5. Is sex work your main source of income? Yes
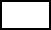
, No
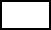

6. Besides sex work, do you have other regular sources of income? Yes
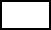
, No
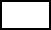

7. Where is your place of sex work located? Urban area
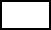
, Rural area
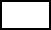
, Both
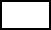

8. Where do you mostly provide sexual services to your clients? (*multiple responses accepted*)

Brothel
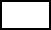
, Bars
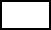
, Night Clubs
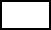
, Street
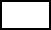
, Own place
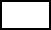
, Client’s place
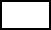
, Others
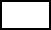
 (specify)

1. Has your client ever refused to pay you after sex work? Yes
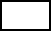
, No
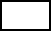

2. Have you ever been verbally abused (insulted) by any of your clients?

Yes
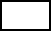
, No
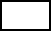


1. Have you ever been physically abused/beaten by your client? Yes
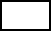
, No
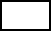

2. Have you ever been forced by your client to have sex (raped)? Yes
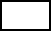
, No
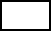

3. Did you use a condom during your last sexual encounter? Yes
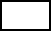
, No
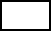

4. How often do you use a condom with your clients?

Always
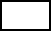
, Sometimes
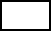
, Never
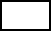


1. Have you ever heard about PrEP? Yes
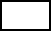
, No
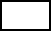

2. Are you currently using PrEP as a method of HIV prevention? Yes
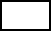
, No
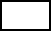

3. Have you ever been arrested by a law enforcer/police for sex work? Yes
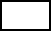
, No
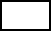

   - 1. **Other factors associated with depression**
4. Do you currently use any of the following illicit drugs? (*multiple responses accepted*)

Shisha
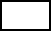
, Marijuana
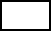
, Mairungi
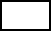
, Injecting drugs
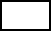


1. How often do you drink alcohol? Always
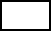
, Sometimes
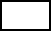
, Never
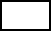

2. In the past 1 week, how many days in the week did you take alcohol?
3. Do you have any long-term physical illness? Cancer
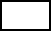
, Hypertension
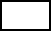
, Diabetes
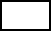
, Tuberculosis
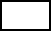
, Hepatitis B
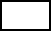
, Others (Specify)
4. Do you know your HIV status? Yes
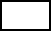
, No
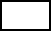

5. If you know your HIV status, what is it? HIV Positive
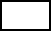
, HIV Negative
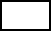

6. If HIV negative, how long ago did you last test for HIV?
7. If HIV positive, are you currently on ARV? Yes
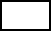
, No
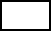

8. If HIV positive, have you disclosed your HIV-positive status to others?

Yes
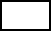
, No
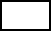


1. Do feel psychologically affected by any significant loss like:

bereavement
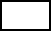
, financial ruin
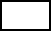
, natural disasters
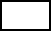
, serious illness or disability
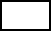
, None of the above
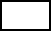

Supplement: Supplementary file 1 — Additional file 1. [file 12889_2021_11207_MOESM1_ESM.docx]
